# Tackling vascular risk factors as a possible disease modifying intervention in Parkinson’s disease

**DOI:** 10.1038/s41531-024-00666-6

**Published:** 2024-03-02

**Authors:** Anne E. Visser, Nienke M. de Vries, Edo Richard, Bastiaan R. Bloem

**Affiliations:** 1 Department of Neurology, Radboud University Medical Center, Donders Institute for Brain, Cognition and Behaviour, Center of Expertise for Parkinson & Movement Disorders, Nijmegen, The Netherlands; 2 Department of Neurology, Haaglanden Medical Center, The Hague, The Netherlands

**Keywords:** Parkinson's disease, Parkinson's disease, Risk factors, Parkinson's disease, Parkinson's disease

## Background

The trialed treatment landscape of Parkinson’s disease (PD) is extensive, but up to now, there is no disease-modifying therapy. As the pathophysiology of PD becomes untangled further, new targets for intervention may arise. Substantial evidence points to alpha-synuclein as a possibly important pathogenic protein in this disease, making it a therapeutically interesting target^1^. However, just as we have seen in the field of Alzheimer’s disease, where targeting the most prominent pathological deposits (amyloid) yielded disappointing results^2^, the mechanisms contributing to neurodegeneration in PD are complex and extend well beyond alpha-synuclein aggregation. Indeed, several recent trials that specifically targeted alpha-synuclein showed no signs of a disease-modifying effect on either clinical scales or imaging outcomes^3^, casting doubt on the viability of an anti-alpha-synuclein-only approach^4^. The question arises whether targeting a single pathogenic target will ever be sufficient to modify the course of PD and whether a complementary, more multifaceted, and comprehensive therapeutic approach would be more effective.

Here, we propose additional therapeutic options from a different angle, namely targeting risk factors that contribute to cerebral small vessel disease (SVD), including lacunar infarcts and white matter lesions. PD and SVD are both common conditions and their incidence increases with age. Importantly, SVD is commonly seen in neuroimaging in PD. Such vascular lesions may be identified during the first assessment in de novo patients, but appear more commonly later on in the course of the disease^5,6^. Both the severity and progression of SVD have been independently associated with incident parkinsonism^7^. When SVD is present in PD, it negatively impacts the clinical symptoms of PD. This includes a worsening of gait, cognition, and mood, and it may well be associated with a further hastening of the already progressive course of PD^6,8^. The severity of SVD correlates with the Hoehn and Yahr motor score^9^.

Several disease mechanisms may underlie the interaction between SVD and PD. The first option is structural SVD lesions located in strategic brain regions, e.g. the basal ganglia. A second option is hypoperfusion in cerebral small vessels. Both of these mechanisms may result in widespread dysfunction of multiple brain pathways, including the disruption of dopaminergic and non-dopaminergic pathways involved in the pathophysiology of motor and non-motor symptoms in parkinsonism^5,10^. Third, there is increasing evidence that the permeability of the blood–brain barrier is increased in SVD. Strikingly, alterations in the blood–brain barrier have also been observed in mesencephalic regions (including the substantia nigra) of patients with PD. This might represent a mechanistic link^10–12^. Specifically, blood–brain barrier dysfunction disrupts astrocyte integrity, which impairs interstitial fluid exchange and decreases neuronal energy supply. It also stops oligodendrocyte precursor cells from maturation, which impairs the formation and repair of myelin and energy support to axons^11^. Fourth, cerebral hypoperfusion could also induce alpha-synuclein aggregation leading to PD pathology with subsequent soluble alpha-synuclein depletion^13,14^.

We hypothesize that the course of PD might be slowed down by an individually tailored and multicomponent intervention, targeting vascular, and lifestyle-related risk factors for SVD. The optimal target group for such an intervention would be patients with PD and radiologically proven SVD (defined as small subcortical (lacunar) infarcts (of deep gray nuclei and deep white matter), microhemorrhages, diffuse white matter changes, and enlarged perivascular spaces). Possible target risk factors are diabetes mellitus, hypertension, dyslipidemia, overweight, unhealthy diet, physical inactivity, sleep apnea, and smoking (Table 1). A combined pharmacological and non-pharmacological approach could be used.

**Table 1:** Risk factors for comorbid cerebral small vessel disease in Parkinson’s disease

Risk factor	Possible relation with PD risk?	Target in multidomain intervention?
Diabetes mellitus	Yes	Yes
Hypertension	Yes	Yes, but monitor symptoms of orthostatic hypotension
Dyslipidemia	The evidence on dyslipidemia (and the treatment thereof) is equivocal	We recommend to await further study results before considering dyslipidemia as a target risk factor
Overweight	Yes	Yes, but avoid underweight and malnutrition
Unhealthy diet	Yes	Yes
Physical inactivity	Yes	Yes
Sleep apnea	Yes, some evidence	Not yet considered a target risk factor
Smoking	Inverse	Causally linked to numerous other diseases and all-cause mortality. It should therefore be avoided.

## Proposed risk factors

Many vascular risk factors have been studied individually in relation to PD. First, type 2 diabetes mellitus has been identified as a possible risk factor for developing PD and also appears to be associated with a faster disease progression. Currently, several glucagon-like peptide-1 receptor agonists are being investigated in patients with PD as potential disease-modifying treatment^15^. Second, the association between hypertension and PD is more difficult to elucidate since PD can be accompanied by orthostatic hypotension, but also by supine hypertension as a compensatory strategy to minimize the consequences of excessive drops in upright standing blood pressure. Moreover, (orthostatic) hypotension is an important adverse effect of most medications used in PD^16,17^. Orthostatic hypotension is associated with nocturia, impairing sleep quality, and may cause fatigue, syncope, and cognitive impairment. As such, orthostatic hypotension negatively impacts the quality of life^18^. Several studies have found an association between orthostatic hypotension (with or without accompanying recumbent hypertension) and a higher prevalence of white matter lesions on brain scans in persons with PD^19,20^. There is no ‘one-size-fits-all’ recommendation, but one potentially interesting (but hitherto poorly studied) option is to instruct patients to sleep with their bed placed in an anti-Trendelenburg position. This simple non-pharmacological intervention reduces nocturnal polyuria, ascertains a more favorable distribution of body fluids and ameliorates nocturnal hypertension^21,22^. Third, various studies assessed whether dyslipidemia or statin use prior to, or at the moment of diagnosis, is associated with PD risk or, following the diagnosis, with faster progression of cognitive or functioning in PD. The results were conflicting^23–26^, possibly because of differences in study design. High cholesterol lowers the risk of PD and has also been associated with slower PD progression^27^. While statins were proposed as potentially slowing the progression of disease, this relationship could be confounded by the high cholesterol that these patients had. Alternatively, the effectiveness of this intervention may depend on hydrophobicity as hydrophilic statins have been associated with faster PD progression^28^. Although simvastatin was found to have an adverse effect on disease progression^29^, a recent trial showed that lovastatin treatment in patients with early-stage PD was associated with a trend toward a reduced progression of motor symptoms. This intervention is now examined further in a phase 2 trial (NCT03242499)^30^. Taken together, the evidence on dyslipidemia (and the treatment thereof) on both the risk of PD and its subsequent progression is equivocal. We, therefore, recommend to await further study results before considering dyslipidemia as a target risk factor. Fourth, the role of body mass index on the risk of PD has been studied extensively, but the results remain inconclusive^31,32^. The association between body mass index and PD prognosis seems to follow a U-shaped curve, with both low body weight and overweight being detrimental^33–37^. The association of weight loss with faster disease progression might well be confounded by factors such as disease duration, dyskinesias, problems with swallowing, or cognitive problems, all leading to a lower nutritional intake. Therefore, many patients with PD tend to spontaneously lose weight. However, targeting persistent overweight might have some merit. Fifth, observational studies suggest associations between adherence to the Mediterranean diet and a lower risk of developing PD, as well as a slower progression of parkinsonism^38,39^. A few pilot studies with a Mediterranean diet in PD have recently been performed showing encouraging effects on constipation, cognition, and motor symptoms^40–42^. Sixth, there is a fast-growing body of evidence to suggest that engaging regularly in physical activities, including both aerobic exercise as well as a sufficient volume of activities, helps to stabilize motor progression and enhances cognitive performance^43,44^. Moreover, recent work by our own group also demonstrated that regularly engaging in aerobic exercise (three times per week for 30–45 min) was associated with less brain atrophy over time, and with enhanced functional connectivity between the basal ganglia and the cortex in persons with PD^45^. And the very same exercise intervention may also help to slow down the development of SVD, for example via beneficial effects on hypertension or diabetes^46^. Seventh, sleep apnea has been suggested to have an association with a higher risk of developing PD as well as with an increased severity of PD in terms of cognitive dysfunction and motor symptoms^47–49^. However, the evidence is still scarce and the role of sleep apnea as a risk factor for cerebral small vessel disease has not conclusively been proven so far^50,51^. Therefore we do not consider sleep apnea as a target risk factor yet. Finally, smoking is associated with a lower risk of developing PD in various studies, but smoking behavior does not appear to affect the rate of disease progression after the diagnosis has been made^52–55^. Because smoking is also causally linked to numerous other diseases and all-cause mortality, it should be avoided. However, the inverse relation with smoking remains an interesting area for future research. It would be worthwhile identifying which factors explain this inverse relation, such as differences in reward mechanisms, a possible protective effect against environmental toxins through reduced permeability of for example the gut, or via specific components of cigarette smoke that might mediate a protective effect on the risk of developing PD.

While multidomain interventions for cardiovascular risk factors (including a combination of diet, exercise, and vascular risk monitoring) have not been performed in PD yet, such trials have been described for other populations. In a recent Cochrane review on multidomain interventions for the prevention of dementia and cognitive decline, the authors concluded that multidomain interventions cannot prevent dementia, but they may have a small beneficial effect on cognitive functioning in older people^56^. Our hypothesized intervention will be based on current guidelines for cardiovascular risk management, taking into account personal preferences (which will be essential to optimize compliance) as well as individual risk profiles^57^. Pharmacological preventive expenditures are relatively low and medication is often well tolerated by patients, possibly resulting in a cost-effective proposal. Ideally, this should be evaluated in a randomized clinical trial in persons with PD of 50 years or older and with modifiable risk factors for cerebral SVD, and where personalized cardiovascular risk management (when added to usual care) would be compared with usual care alone. Cardiovascular risk management should be individually tailored to the specific risk profile of the participant, targeting different vascular risk factors (i.e. diabetes mellitus, hypertension, overweight, unhealthy diet, physical inactivity, and smoking). The intervention must be guided by health professionals to tailor the intervention and to monitor adherence and dosage. As the associations between some of these risk factors and PD risk and/or progression are complex, professional guidance is warranted. For example, when focusing on losing weight, underweight and imbalanced nutritional status (e.g. lack of proteins) should be avoided. The outcomes of such a trial should primarily be measured clinically, for example using motor and cognitive symptoms as an indication of disease progression.

## Summary

In summary, we hypothesize that the progression of PD might be slowed down by treating common vascular risk factors. This calls for a complex, multifaceted intervention with a personalized approach. This hypothesis now needs to be taken to the test.
